# Morbimortality and Six-Month Survival Among Elderly Patients Treated With Noninvasive Mechanical Ventilation in an Intermediate Care Unit: A Retrospective Evaluation

**DOI:** 10.7759/cureus.32013

**Published:** 2022-11-29

**Authors:** Mafalda F Teixeira, Mónica Lopes, Frederico Batista, Ana Bastos Furtado, José Delgado Alves

**Affiliations:** 1 Department of Internal Medicine IV, Hospital Professor Doutor Fernando Fonseca, Lisboa, PRT

**Keywords:** pneumonia, do-not-intubate order, elderly, acute respiratory failure, noninvasive mechanical ventilation

## Abstract

Background: Noninvasive mechanical ventilation (NIMV) has been established as a successful therapeutic option for patients with acute respiratory failure (ARF) with a specific etiology.

Objectives: This study evaluated the morbimortality of patients with ARF treated with NIMV in a medical intermediate care unit (UCINT) to identify factors associated with higher in-hospital mortality, six-month mortality, and three- and six-month hospital readmission rates.

Methods: This retrospective cohort study included elderly patients admitted for ARF and treated with NIMV in the UCINT between 2015 and 2019.

Results: In the sample of 102 patients, the median age was 84.2 (±5.5) years, and 57% were women. In total, 28% were on long-term oxygen therapy, and 68% had a do-not-resuscitate order. At admission, the median Charlson comorbidity index and Barthel index of activities of daily living were 7 [6; 8] and 30 [20; 57,5], respectively. The simplified acute physiology score II was 39.1±10.7, and 92% of patients had type 2 ARF. Median days on NIMV and days in UCINT were 10 [6; 16] and 6 [3; 10], respectively. The main conditions requiring UCINT admission for NIMV were heart failure, pneumonia, and exacerbation of the chronic obstructive pulmonary disease. The NIMV failure rate was 7%. At discharge, the average Barthel index was 35 [10; 55]. The in-hospital mortality rate was 23%.

Discussion: Older age, higher simplified acute physiology score II, higher Charlson comorbidity index, and higher number of days on NIMV were associated with higher in-hospital mortality. Long-term oxygen therapy was associated with higher three-month mortality. A higher Barthel index at the time of hospital discharge was associated with a higher six-month readmission rate.

Conclusion: NIMV can be used successfully in elderly patients and less studied ARF etiologies, such as pneumonia.

## Introduction

As people around the world live longer, older adults are increasingly developing comorbidities and limitations [1]. For example, the incidence of acute respiratory failure (ARF) increases with age, and elderly patients are particularly susceptible to chronic heart failure and cardiogenic pulmonary edema, pulmonary diseases such as chronic obstructive pulmonary disease (COPD), hypercapnic respiratory failure, and other conditions requiring ventilatory support [2-9]. These patients also often have comorbidities and functional limitations and thus are not candidates for aggressive therapeutic measures, such as endotracheal intubation. Compared to more invasive methods, noninvasive mechanical ventilation (NIMV) can reduce complications associated with intubation and improve hospital survival in these patients [10-14]. Despite some evidence showing the utility of NIMV in an elderly population in respiratory intensive care units [15], specific data regarding the use of NIMV to manage ARF in very old persons is limited [16-19]. The objective of this retrospective study thus was to evaluate morbimortality among patients aged 75 years and older with ARF who were treated with NIMV and to identify factors associated with higher in-hospital and six-month mortality rates as well as higher three- and six-month hospital readmission rates.

This article was previously presented as an oral presentation at the 35º Congresso Nacional de Pneumologia on November 7, 2019 and as a poster at the European Respiratory Society International Virtual Congress in September 2020.

## Materials and methods

Study design

This retrospective observational study was done in the intermediate care unit (UCINT) at Hospital Professor Doutor Fernando Fonseca in Portugal between January 2015 and January 2019. This central hospital near Lisbon provides care for approximately 700,000 people. The UCINT has six beds and is integrated with the internal medicine department to monitor patients’ hemodynamic parameters, such as blood pressure, heart and respiratory rates, peripheral oxygen saturation, and electrocardiogram. The UCINT provides NIMV using Vivo 30 Bi-Level ventilators. The nurse-to-patient ratio is 1 to 6, and at least two internists are on duty during the morning shift, with an internist on call on evenings and weekends.

Study population

Patients aged 75 and older who were admitted to the UCINT between January 2015 and December 2018 and underwent NIMV for acute or acute-on-chronic respiratory failure were admitted to the study. Exclusion criteria included NIMV for palliative care, to wean from invasive mechanical ventilation, and for respiratory issues caused by a neurologic disease (e.g., amyotrophic lateral sclerosis), as well as those with ambulatory use of NIMV and those missing adequate clinical data.

Data retrospectively collected from the clinical files included age, gender, ambulatory living facility, Barthel and Charlson scores, need for long-term oxygen, and presence of do-not-intubate order. ARF etiology was classified according to disease groups as follows: decompensated heart failure, COPD exacerbation, pneumonia, sleep apnea/hypopnea syndrome, other pulmonary diseases (e.g., interstitial lung disease), postoperative, and other (e.g., benzodiazepine intoxication). The simplified acute physiology score II and blood gas (pH, pCO_2_, pO_2_, HCO_3_) levels before beginning NIMV also were collected.

In-hospital variables included the department where NIMV was started, number of days admitted before starting NIMV, duration of NIMV in days, and NIMV failure (defined as clinical and gasometric deterioration leading to orotracheal intubation in patients without a do-not-intubate order). Do-not-intubate orders were decided upon admission to the UCINT according to the best clinical judgment. Length of hospital stay (days), length of stay in the UCINT (days), physical rehabilitation, and in-hospital mortality also were collected.

Data at discharge included Barthel's score and whether the patient received ambulatory NIMV or long-term oxygen therapy. Hospital re-admissions and deaths within one, three, and six months also were evaluated.

Statistical analysis

Categorical variables are expressed as absolute and relative frequencies. Continuous variables are described as means and standard deviation for normal distributions and median and interquartile ranges otherwise. To test hypotheses in groups of equal size, we used the t-test for independent groups and analysis of variance with one factor when it was appropriate to assume a normal distribution of variables. Testing of the non-normal distribution of continuous variables was performed using a non-parametric Mann-Whitney U or Kruskal-Wallis test, depending on the hypothesis being tested. Significance was set at p = 0.05. Data analysis was performed using the Statistical Package for Social Sciences version 14.0 (IBM, Armonk, NY).

## Results

**Figure 1:**
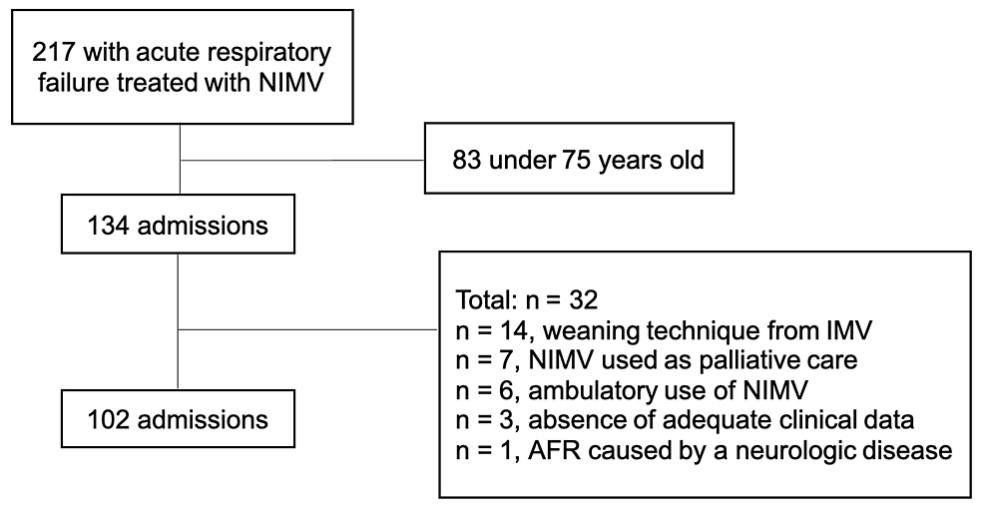
Admission and exclusion criteria NIMV = noninvasive mechanical ventilation, IMV = invasive mechanical ventilation, ARF = acute respiratory failure

Out of 217 patients treated with NIMV in the UCINT during the study period, 102 were included (Figure 1), 58 (57%) of whom were female. The mean age was 84.2 years (±5.5), with a maximum age of 96. Although only 13% lived in nursing facilities before hospital admission, patients were overall very dependent, as shown by a mean Barthel index score at the admission of 30 (20; 57.5). The mean Charlson comorbidity index at admission was 7 (6; 8), indicating an overall estimated 10-year survival probability of 0%. Although 94 (92%) participants presented with hypercapnic respiratory failure, most (68.1%) were not eligible for intubation. In addition to NIMV, 29 (28%) also were prescribed long-term oxygen therapy. Arterial blood gas values at the beginning of NIMV were as follows: pH of 7.3±0.08 and PaCO_2_ of 64.2±16.7. Table 1 summarizes the results.

 

**Table 1:** Patient’s characteristics at enrolment DNI order = do-not-intubate order, LTOT = long-term oxygen therapy

Characteristics	
Age	84,2±5,5
Female sex	58 (57%)
Living in nursing facility	13 (13%)
Comorbidities (Charlson index)	7 [6; 8]
Barthel Index for Activities of Daily Living	30 [20; 57,5]
LTOT	29 (28%)
DNI order	62 (68.1%)

NIMV was initiated in the emergency department for 66% of participants, in the UCINT for 27%, and in other hospital departments for the rest. For patients whose NIMV was initiated in the UCINT, the mean time until starting NIMV was 3 [0; 4] days. Upon admittance to the UCINT, the average simplified acute physiology score II was 39.1±10.7, with a probability of in-hospital mortality of 23%. The main causes of ARF were decompensated heart failure (79%), pneumonia (49%), COPD exacerbation (33%), and sleep apnea/hypopnea/hypoventilation obesity syndrome (19%). Table 2 summarizes the results.

**Table 2:** Main reasons to begin NIMV NIMV = noninvasive mechanical ventilation, COPD = chronic obstructive pulmonary disease, SAHS = Sleep apnoea/hypopnoea syndrome

Main reasons to begin NIMV	Number of patients	Total, %
Decompensated heart failure	81	79
Pneumonia	50	49
COPD exacerbation	34	33
SAHS	19	19

More than half (62%) of patients underwent respiratory physiotherapy while in the UCINT. Median days on NIMV were 6 [3; 10] in the unit and 10 [6; 16] in total, with a 7% rate of therapeutic failure. The mean hospital stay was six days in UCINT and 18 in total. The mean Barthel index at time of discharge was 35 [10; 55]. Upon discharge, 33 (41%) were prescribed long-term oxygen therapy and 36 (44%) were prescribed NIMV. Regarding mortality rates, 21 (21%) patients died in the hospital and 23% within six months of discharge. Among those who were readmitted to the hospital, 25% were readmitted within one month of discharge, 27% within three months, and 17% within six months. Most (81%) readmissions within three months were for the same health issue, and 71% needed NIMV.

The following factors were found to be associated with higher in-hospital mortality: higher age, higher simplified acute physiology score II, and longer duration to starting NIMV (see Tables 3, 4). We found no factors associated with a higher hospital readmission rate or mortality within three months. At six months after discharge, the mortality rate had a marginally significant (p=0.053) relation to the Barthel index score at discharge. Table 5 summarizes the results.

**Table 3:** Univariable logistic regression models for predictors of in-hospital mortality *Not used in the multivariate analysis due to the low number of patients with sepsis (cohort vs. dead: 1/81 (1%) / 3/21 (14%)). NIMV = noninvasive mechanical ventilation, UCINT = medical intermediate care unit, LTOT = long-term oxygen therapy

Univariable logistic regression model	P-value	OR
Age	0,034	1,11 (CI: 1,01; 1,22)
Gender	0,15	
LTOT	0,29	
Charlson index	0,25	
Barthel index for activities of daily living	0,95	
SAPS II	0,009	1,07 (CI: 1,01; 1,12)
Heart failure	0,31	
COPD	0,80	
Pneumonia	0,07	
SAHS	0,23	
Sepsis	0,027*	
pH value at beginning of NIMV	0,90	
pCO2 value at beginning of NIMV	0,51	
pO2 value at beginning of NIMV	0,81	
HCO3 value at beginning of NIMV	1,49	
Time until beginning NIMV	0,001	1,15 (Cl: 1,03; 1,27)
Beginning NIMV at UCINT	0,056	
Duration of NIMV (total)	0,89	
Duration of NIMV (UCINT)	0,17	
Length of hospital stay (total)	0,66	
Length of hospital stay (UCINT)	0,056	

**Table 4:** Multivariable logistic regression models for predictors of in-hospital mortality NIMV = noninvasive mechanical ventilation

Multivariable logistic regression model	P-value	OR
Age	0,028	1,13 (CI: 1,01; 1,27)
SAPS II score	0,032	1,06 (CI: 1,01; 1,12)
Time until beginning NIMV	0,042	1,12 (CI: 1,00; 1,26)

 

**Table 5:** Univariable logistic regression models for predictors of six-month mortality NIMV = noninvasive mechanical ventilation, LTOT = long-term oxygen therapy

Univariable logistic regression model	P-value
Age	0,14
Gender	0,79
LTOT at time of discharge	0,82
NIMV at time of discharge	0,79
Charlson index at time of discharge	0,55
Barthel index at time of discharge	0,053
Heart failure	0,31
COPD	0,31

## Discussion

To our knowledge, this is one of the first studies to assess the efficacy and safety of NIMV in elderly patients in a Portuguese intermediate care unit, where more than two-thirds of patients admitted have ARF. Other studies and the main guidelines on NIMV cite pneumonia as the primary motive for beginning NIMV. In our study, the primary diagnosis was decompensated heart failure. This discrepancy may be because most (68%) patients treated with NIMV in the UCINT during the study period had a do-not-intubate order and therefore were not candidates for invasive mechanical ventilation. However, over the last decade, the use of NIMV has significantly increased in patients with pneumonia, despite mixed evidence regarding its efficacy in preventing intubation [20,21]. In a study conducted by Stefan et al. comparing outcomes among 3,971 patients hospitalized with pneumonia and treated with noninvasive versus invasive mechanical ventilation, initial NIMV was associated with better survival among the subgroup of patients hospitalized with pneumonia who had COPD or heart failure [22]. Patients treated with NIMV were older, and NIMV failure was more common among patients without COPD or heart failure comorbidity. Whereas in Stefan et al.'s study most patients admitted with pneumonia did not have a previous diagnosis of COPD or heart failure, almost all participants in our study had hypercapnic respiratory failure, suggesting they probably had undiagnosed comorbidities that contributed to the development of this type of respiratory failure.

NIMV failed in seven (7%) participants in this study, similar to rates reported by Nava et al., in which 7.3% of patients over the age of 75 with respiratory failure who required NIMV had to be intubated [12]. The mean simplified acute physiology score II at admission to UCINT was 39.1, and the in-hospital (six-month) mortality rate was 21% (23%). In another study published by the Scarpazza group, in-hospital mortality was only 13% [18]. However, in that case, the main cause for admittance was COPD exacerbation, and no patients with heart failure were recruited. Calvo and colleagues, whose sample had a similar distribution of conditions leading to NIMV, reported a similar in-hospital mortality rate [23].

According to our multivariate analysis, in-hospital mortality is associated with older age (p=0.028; odd ratio=1.13) and a higher simplified acute physiology score II at UCINT admission (p=0.032; odd ratio=1.06), in line with other studies. We also found evidence of a link between mortality and the length of time between admission and beginning NIMV (p=0.042; odd ratio=1.12). This result suggests that delaying the start of NIMV may be deleterious and that a lower threshold for initiating NIMV may be warranted in these patients. In a study of patients with COPD and ARF, Nava et al. similarly concluded that more severe ARF is a likely cause of NIMV failure [1].

We found some evidence of a relationship between the six-month mortality rate and the Barthel index score at discharge (p=0.053). This finding may reflect the impact of other comorbidities on quality of life, rather than just respiratory dysfunction.

We found that 25% of participants were readmitted within one month of discharge, 27% within three months, and 17% within six months. These results are comparable with those of Calvo et al., who found that compared to younger patients, those over 75 years old had a greater tendency toward rehospitalization and need for NIMV in the 6-12 months after discharge [23]. Elderly patients may have lower functional status at discharge, which could increase their vulnerability toward rehospitalization. Most (81%) readmissions within three months were due to the same health issue, and most (71%) needed NIMV again. This observation is consistent with the presence of chronic conditions underlying respiratory failure and the consequent need for NIMV. Future studies should evaluate the quality of life in elderly patients who require NIMV after discharge, because a poorer quality of life may lead to rehospitalization.

We note several limitations of our study. First, this retrospective and observational research may be prone to bias. Second, the relatively small number of patients limits the generalizability of the results. Third, there is a lack of relevant data to explain NIMV failures, such as the existence of delirium, difficulty clearing bronchial secretions, or the need to use a mechanical insufflation-exsufflation device.

## Conclusions

In this study, we analyzed the administration of NIMV in elderly patients with ARF who was admitted to a hospital intermediate care unit. The results from the data, which span three years, support the idea that NIMV can be used successfully in the elderly, even in less well-studied acute respiratory insufficiency etiologies like pneumonia. In our cohort, the in-hospital mortality rate was similar to that described in the literature for specific respiratory units. Delaying the start of NIMV was identified as a factor associated with higher in-hospital mortality. Also, deterioration of the patients’ overall health during their hospital stay was associated with long-term mortality. Overall, the findings highlight the importance of a holistic approach to these patients.
